# Gas chromatography-Mass Spectra analysis and deleterious potential of fungal based polythene-degradation products

**DOI:** 10.1038/s41598-018-37738-6

**Published:** 2019-02-07

**Authors:** Manisha K. Sangale, Mohd. Shahnawaz, Avinash B. Ade

**Affiliations:** 1 Department of Botany, Savitribai Phule Pune University, Pune, 411007 Maharashtra India; 2 Present Address: Department of Botany, S M Joshi College Hadapsar, Malwadi, Hadapsar, Pune, Maharashtra 411028 India; 3 Present Address: Plant Biotechnology Division, CSIR-Indian Institute of Integrative Medicine, Canal Road Jammu, Jammu, 180001 Jammu and Kashmir India

## Abstract

Polythene-degradation products (PE-DPs) produced due to two most efficient polythene degrading fungal isolates (*Aspergillus terreus* strain MANF1/WL and *Aspergillus sydowii* strain PNPF15/TS) after 60 days of incubation at ambient temperature with continuous shaking were analyzed by employing GC-MS method. Total 24 PE-DPs were recorded in total 4 samples i) control (pH 3.5), ii) Treatment of *Aspergillus terreus* strain MANF1/WL (pH 3.5), iii) control (pH 9.5) and iv) Treatment of *Aspergillus sydowii* strain PNP15/TS (pH 9.5). To check the deleterious status of PE-DPs using both the elite fungal isolates at *in vitro* level, two living systems (Sorghum and Tiger shark) were used. The percent germination rate of sorghum seeds were found unaffected with PE-DPs of both elite fungi. PE-DPs of both the fungal isolates exhibited maximum germination index at 50%. Whereas, highest elongation inhibition rate (34.75 ± 7.10) was reported with PE-DPs of *Aspergillus terreus* strain MANF1/WL. In case of animals system, no mortality of the Tiger sharks was documented after fifteen days of the treatment.

## Introduction

Based on various beneficial properties, plastic is one of the widely used polymers throughout the world, Due to its luxuriant usage, billion tons of plastic waste is being generated into the environment annually^1,2^. Polythene represents 64% of the total plastic waste. Plastic waste leads to various problems at both terrestrial and marine environment. At dumping site, plastic is being eaten by the terrestrial animals along with the food stuff. The plastic remains undigested and leads to the death of various animals^3^. Most of the plastic waste finally reaches to the marine environment and leads to the death of billions of marine animals due to intestinal blockage, and their entanglement with the plastic^4^. The plastic gets degraded under natural condition with very slow speed and took few hundred years to mineralize. To tackle with this emerging issue of the plastic waste management, various researchers around the globe tried to degrade the plastic with various methods^1,2^ and reported biodegradation as the most safe, widely accepted and environment friendly method^5^. We also attempted to isolate, screen and characterize the polythene degrading fungi from the rhizosphere soil of *Avicennia marina* growing along the West Coast of India. We reported two elite polythene degrading fungal isolates (*Aspergillus terreus* strain MANF1/WL and *Aspergillus sydowii* strain PNPF15/TS) based on percent weight loss, percent loss in tensile strength, scanning electron microscopy (SEM) and Fourier transform infra-red spectroscopy (FTIR) after 60 days of continuous shaking at ambient temperature (unpublished). In the present investigation, in order to understands the deleterious nature of by-products produced due to action of these fungal isolates, firstly we tried to identify the polythene degraded by-products by employing GC-MS tool, followed by assessing their deleterious potential effects on Sorghum (plant system) and tiger shark (animal system) at *in vitro* level.

## Results

### Investigation of PE-DPs formed by the fungal isolates using GC-MS analysis

**Figure 1:**
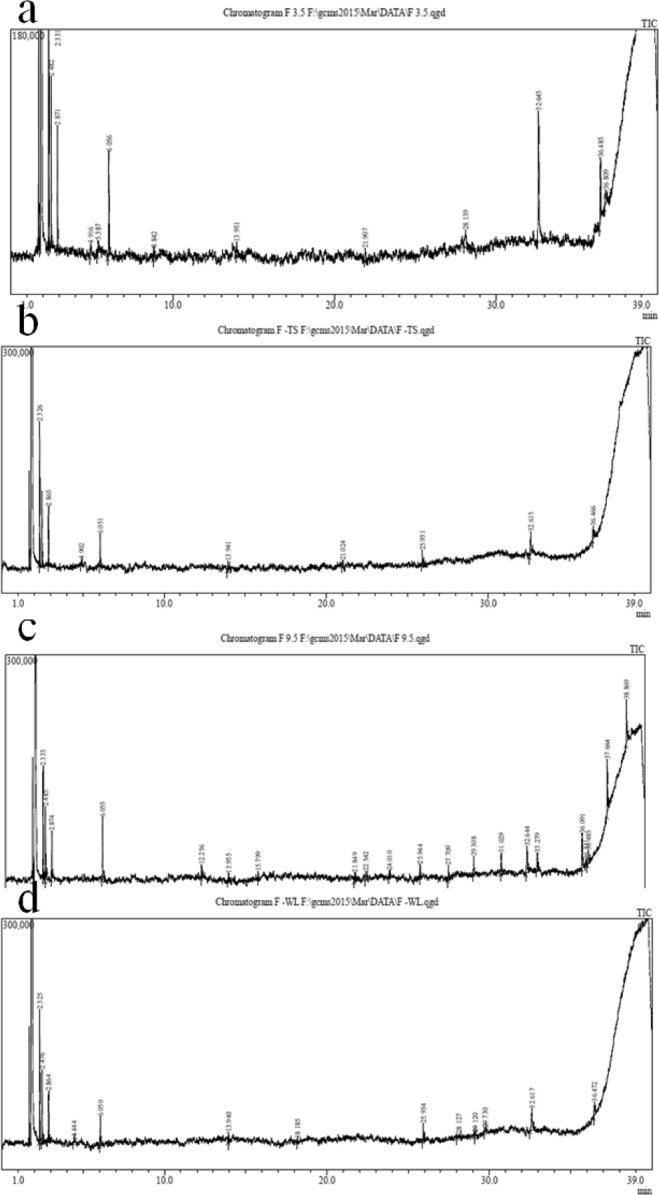
Chromatogram of polythene-degradation products obtained from: (**a**) control PE at pH 3.5; (**b**) *Aspergillus sydowii* strain PNPF15/TS (pH 3.5); (**c**) control PE at pH 9.5 and (**d**) *Aspergillus terreus* strain MANF1/WL (pH 9.5).

The by-products of polythene strips produced by the two elite fungi isolates (*Aspergillus terreus* strain MANF1/WL and *Aspergillus sydowii* strain PNPF15/TS) growing in Sabouraud dextrose broth with continuous shaking at ambient temperature were documented using GC-MS analysis. Total 24 by-products were recorded in total 4 samples i) control (pH 3.5), ii) Treatment of *Aspergillus terreus* strain MANF1/WL (pH 3.5), iii) control (pH 9.5) and iv) Treatment of *Aspergillus sydowii* strain PNP15/TS (pH 9.5) (Fig. 1; Supplementary Table S1). Four byproducts such as Trimethylsilyl methanol, Propane, Pentanoic acid, 2-Butoxyethylacetate was found in all the samples. In case of PE-DPs of the control at pH 3.5 and 9.5, total 9 and 10 by-products were recorded respectively. Among them 6 by-products were found common in both (pH 3.5 and 9.5). Due to action of *Aspergillus sydowii* strain PNPF15/TS total 10 by-products were produced. Among them 6 were similar to control and only 4 were unique. These were 7- Methylenebicyclo [3.2.0] hept-3-en-2-one; Dibutyl phthalate; 1,4-Benzenediol and Dodecahydropyrido [1,2-b] isoquinolin-6-one at retention time (RT) 4.90, 21.02, 28.01 and 28.45 respectively. Total eleven by-products of polythene degraded with *Aspergillus terreus* strain MANF1/WL were recorded; among these 11 by-products 5 were similar with that of control and 6 different by-products were formed, 2 Naphthalene carboxylic acid; Dibutyl phthalate; 2-Cyclohexen; 1,2-Bis (Trimethylsilyl) benzene; Hexasiloxane and Hexadecanoic acid.

### Assessment of deleterious potential of polythene-degradation products (PE-DPs)

#### Deleterious potential effects of PE-DPs on Sorghum

**Figure 2:**
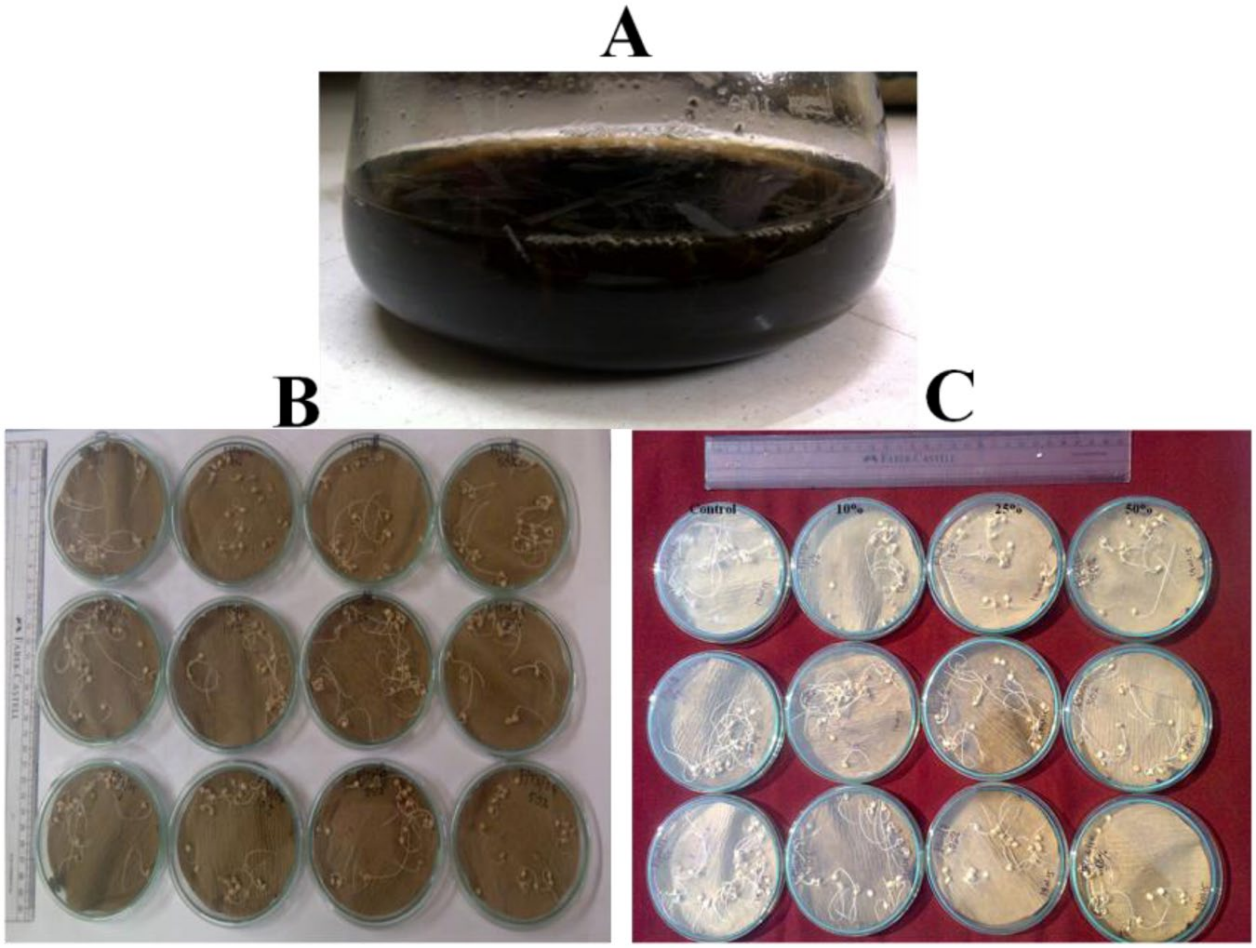
Effect of PE-DPs on sorghum seeds. (**A**) Flask with PE-DPs produced by fungal isolates after 60 days of continuous shaking at ambient temperature. (**B**) Deleterious effect of PE-DPs on sorghum seeds produced due to action of *Aspergillus terreus* strain MANF1/WL; (**C**) Deleterious effect of PE-DPs on sorghum seeds produced due to action of *Aspergillus sydowii* strain PNPF15/TS.

Deleterious potential effects of PE-DPs on percent seed germination: Highest percentage of seed germination of the sorghum seeds (91.67 ± 7.64) were recorded at 25% of PE-DPs by *Aspergillus terreus* strain MANF1/WL (Fig. 2; Supplementary Table S2). The difference in germination percentage of Sorghum seeds with increasing concentration of PE-DPs was found non-significant at 0.05 level of significance based on Duncan test of variance.

Deleterious potential effects of PE-DPs on percent elongation inhibition rate: With the increasing concentration of PE-DPs the elongation inhibition rate of Sorghum seeds was found to follow ascending order (Supplementary Table S3). The elongation inhibition rate with *Aspergillus terreus* strain MANF1/WL at 25 and 50% of PE-DPs was found significantly different from the results at 10% of PE-DPs concentration. Whereas *Aspergillus sydowii* strain PNPF15/TS based polythene by-products were not found to affect the elongation inhibition rate significantly.

Deleterious potential effects of PE-DPs on germination index: The highest value of germination index of the Sorghum seeds (GI%: 112.27 ± 25.04) was recorded at 25% of the concentration PE-DPs produced with *Aspergillus sydowii* strain PNPF15/TS (Supplementary Table S4). At 10% of PE-DPs of *Aspergillus terreus* strain MANF1/WL least germination index was recorded.

Overall, the percent seed germination, germination index and percent elongation inhibition rate of the Sorghum at the selected concentrations of PE-DPs of both the fungi was of moderate order. All the Sorghum seeds survived after the treatment.

#### Deleterious potential effects of PE-DPs on tiger shark

**Figure 3:**
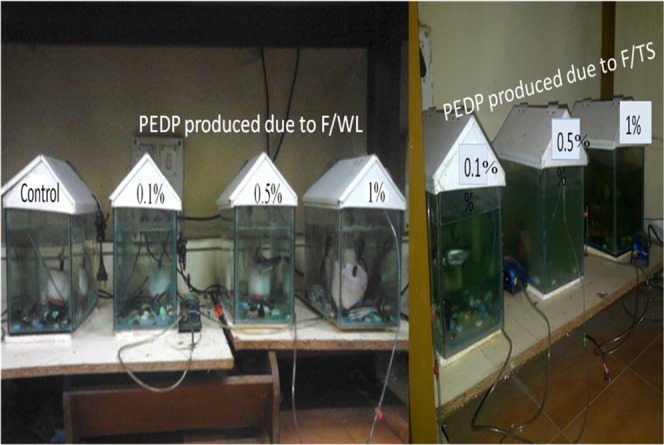
Laboratory set up of the deleterious/toxicity testing of fungal based PE-DPs on tiger shark.

No death of the tiger shark was recorded (Fig. 3; Supplementary Table S5) at any concentration.

## Discussion

The possible by-products generated due to bio-degradation process of polythene were recorded using GC-MS analysis. *Aspergillus* and *Penicillium* reported to produce Octadecanoic Acid, Octadecatrionic Acid, Benzene Dicarboxylic Acid, Cyclopropane, Butanoic Acid as by-products of polythene^6^. As per reports^7,8^, due to abiotic degradation of polymer, either due to thermal oxidation or due to presence of plastic degrading enzyme *viz*. oxidoreductase, Ester- and keto-carbonyls were recorded as the major by-products. In our previous study^9^, we reported total 4 major kinds of by-products of polythene (degraded by the bacterial isolates).

In the present investigation, the dissociation rate of fungal treated PE was found to be higher than the untreated PE. PE (20micron thick) strips treated with *Aspergillus terreus* strain MANF1/WL (pH 9.5) results in the formation of some key compounds viz. 2 Naphthalene carboxylic acid and Dibutylpthalate, 1,2-Bis(trimethylsilyl) benzene, Hexasiloxane, 2-Cyclohexen-1. It indicates more degradation of PE films with the treatment of *Aspergillus terreus* strain MANF1/WL. Similarly the occurrence of Diethyl phthalate, 1,2 Benzenedicarboxylic acid, 7-Methylenebicyclo [3.2.0] hept-3-en-2-one, Octadecanoic acid, Cyclohexane-1, Dodecahydropyrido [1,2-b] isoquinolin-6-one, 1,4-Benzenediol in PE treated by *A. sydowii* strain PNP15/TS dipicts more degradation than control at pH 3.5. The occurrence of peak area becomes more or less due to formation or deformation of polythene compounds generally depends on the composition of the media used for fungal growth and the deleterious potential of the fungi. The occurrence or separation of plasticizer (Dibutyl phthalate) in the polythene degrading product is due to the action of fungal treatment by *A. sydowii* strain PNP15/TS. Similarly the formation of 1,2 Benzene dicarboxylic acid (that is Phthalic acid) is also the indication of polythene degradation by-product which is less toxic. The formations of 7- Methylenebicyclo [3.2.0] hept-3-en-2-one belong to ketones and toxic activity is not reported^10^.

Based on the type of the tested organism and targeted possible toxic by-products, various kinds of toxicity testing methods were approved by different regulatory commissions in different countries throughout the world. Paabo and Levin^11^ reported five major testing methods to estimate the toxicity /deleterious potential of the by-products formed due to both biotic and abiotic degradation of polymers on rats. In case of plant system, various germination indices *viz*. percent seed germination, germination index, percent inhibition elongation rate etc. can be used to determine the deleterious effect of toxic compounds^12^. In the current investigation, the deleterious effect of the fungal based degradation by-products of polythene on the Sorghum seeds was studied using three key germination indices (percent seed germination, percent elongation inhibition rate and germination index) after completing 6 days of incubation at ambient temperature. The percentage of Sorghum seed was found to be varied from 84.33 ± 2.89 (PE-DPs of *Aspergillus terreus* strain MANF1/WL at 50%) to 95 ± 8.66 (Control) but the variation in percent seed germination was found non-significant at all the three concentrations at 0.05 level of significance. The maximum percent elongation inhibition rate (34.75 ± 7.10) of the sorghum seeds were reported with *Aspergillus terreus* strain MANF1/WL at 50% concentration of PE-DPs. Whereas maximum germination index of the Sorghum seeds (39.66 ± 13.94) were recorded with PE-DPs of *Aspergillus sydowii* strain PNPF15/TS. Overall the PE-DPs of both the elite fungal isolates, exerts moderate toxicity effect on plants and no/least toxicity on animals system. Aswale^13^ studied toxicity of polythene-degradation products of both bacteria and fungi and documented moderate effect on rate of seed germination of various tested plant seeds.

Das and Kumar^14^ assessed the toxicity of polythene-degradation products (PE-DPs) formed by using two different bacterial strains *Bacillus* sp.1 and *Bacillus* sp.2 on two different types of plant seeds *viz*. *Cicer arietinum* (recorded maximum percent seed germination: 60%) and *Vigna radiate arietinum* (recorded maximum m percent seed germination: 80%). They reported hampered shoot growth in *C. arietinum* and *V. radiate* due to PE-DPs of *Bacillus* sp.1 and *Bacillus* sp.2 respectively. In agreements with the previous studies we also reported increment in root length of the Sorghum as compared to control with the PE-DPs of both the fungal isolates. Lithner^15^ enlisted several different methods used to assess the toxicity of by-products of polythene on test animals and suggested ‘acute toxicity method’ as the most simple and shortest method, whereas ‘chronic toxicity testing method’ is regarded as the most sensitive, environmentally feasible method and requires more time for toxicant exposure at low concentration. Previously some workers have reported the toxicity effect of different kinds of plastic by-products on various animals e.g. mud snails feeding on the PET mineral bottles, recorded failure of endocrine sytem^16^. To test the toxicity of different kinds of plastic products leachates on animals. As per report^15^, acute toxicity was reported in 30% test samples of *Daphnia magna* due to plastic products leachates. The ‘acute toxicity’ methods have various shortcomings as it is unable to diagnose other deleterious effects on animals. So, it is not easy to determine the non-hazardous potential of the by-products of the polythene degradation based on this test, it can be used at preliminary level for screening purposes. In the current investigation we selected the tiger shark (due to availability in the market) for testing the potential deleterious effects of PE-DPs produced by *Aspergillus* *terreus* strain MANF1/WL and *Aspergillus sydowii* strain PNPF15/TS and we did not recorded any death of the fish after 15^th^ day of the treatment. Aswale^13^ used *Chironomous* larvae for testing the toxicity of PE-DPs produced by the action of microbes along with the filtrates and documented non-significance level of deaths.

## Material and Methods

### Investigation of PE-DPs formed by the fungal isolates using GC-MS analysis

The polythene degrading fungi, *Aspergillus terreus* strain MANF1/WL and *Aspergillus sydowii* strain PNPF15/TS were used for polythene degradation *in vitro* by inoculating with the polythene strips (20 µm) in the Sabourauds dextrose broth. After 60 days of incubation with continuous shaking at ambient temperature, only the PE-DPs produced by most efficient polythene degrading fungi were subjected to GC-MS analysis. After removal of polythene pieces, the fungal suspension was filtered using filter paper (Whatmann no. 2). The culture filtrate contained the polythene degrading products was centrifuged at 8000 rpm at ambient temperature for five minutes^14^. The supernatant was transferred to fresh vial. From the vial 10 ml of supernatant was solubilized in 10 ml of diethyl ether and the polythene-degrading products (PE-DPs) dissolved in diethyl ether was separated using separating funnel. For the GC-MS analysis, 1 ml of the PE-PDs dissolved in diethyl ether was injected in GC-MS (GC-MS-QP2010 Ultra Gas Chromatograph Mass Spectrometer, Shimadzu) and Helium (He) was utilized as a carrier gas. GC-MS analysis was carried out at Central Instrumentation Facility Center, Savitribai Phule Pune University (SPPU), Pune. The major important peaks of the chromatograms were identified based on the library search using GC-MS Solution real time analysis.

### Assessment of deleterious potential of fungi based polythene degrading products (PE-DPs)

#### Deleterious potential effect of fungi based PE-DPs on Sorghum

For the assessment of deleterious potential of polythene degrading products on Sorghum seeds [HY Forage Sorghum Variety MFSH-4 (Raseela), Maharashtra Hybrid Seed Company Limited Mumbai] were selected and purchased from the Swargate area of Pune city, Maharashtra, India. Sorghum seeds were surface sterilized with 4% Sodium hypochlorite solution^17^. Seed germination assay was assessed in Petri plates at ambient temperature. For this purpose, Petri plates along with germination paper were autoclaved at 120 lb pressure for twenty minutes. After autoclaving twenty sterilized seeds were placed in each Petri plates under aseptic conditions. The supernatants of the elite fungal isolates, based on percent weight loss, percent loss in tensile strength, SEM and FTIR analysis were diluted to 10%, 25% and 50%. All the experiments were performed in triplicates for each concentration of PE-DPs solutions. From each concentration 2 ml solution was added in each plate at an alternate day. For the control plate, 2 ml of distilled water was sprinkled. All the Petri plates of the germination assay were incubated under dark condition at ambient temperature. After completion of sixth day of incubation, germination percentage, elongation inhibition percentage and germination index were calculated using the below formulae^18,19^:

$${\rm{Percent}}\,{\rm{Germination}}\,{\rm{rate}}\,( \% {\rm{G}})=\frac{No.of\,seeds\,germinated}{total\,no.\,of\,seeds}\times 100$$

$${\rm{Percent}}\,{\rm{Elongation}}\,{\rm{inhibition}}\,{\rm{rate}}\,( \% {\rm{EI}})=\frac{Lc-Ln}{Lc\,}\times 100$$

Lc: total length of the root in control, Ln: total length of the root of the treated seed

$${\rm{Germination}}\,{\rm{Index}}({\rm{GI}})=\frac{Gn\,\times Ln}{Gc\times Lc}\times 100$$

Gn: average percent seed germination in treated, Ln: average length of the root in treated,Gc: average percent seed germination in control, Lc: average length of the root in control.

#### Deleterious potential effect of fungi based PE-DPs on animal system

To test the toxicity of PE-DPs on animals, Tiger sharks fish was selected, due to their availability in the fish market. Fishes were purchased from the fish market, Aundh, Pune, Maharashtra. Survival of the fishes were checked for one month in the aquarium by feeding them once in a day and changing water of the aquarium after 15 days and then were used for the experimental purpose.

Same PE-DPs solutions were used for toxicity testing in the plant system. In case of animal system, these PE-DPs solutions were diluted as 0.1%, 0.5% and 1% for the treatment. Similar to plant system, for the treatment and control set, five days old water was used for avoiding shock to the fishes. For the % loss in TS set, three aquarium having three fishes in each aquarium along with control and for the set based on % WL same trend was followed using the common control. Additions of diluted PE-DPs solution was done on the first day of experiment and viability of fishes were recorded every day up to the 15^th^ day of experiment. After 15th day of the treatment, the mortality percentage of the tiger shark fishes was calculated by using following formula:

$${\rm{M}}{\rm{o}}{\rm{r}}{\rm{t}}{\rm{a}}{\rm{l}}{\rm{i}}{\rm{t}}{\rm{y}}\,{\rm{p}}{\rm{e}}{\rm{r}}{\rm{c}}{\rm{e}}{\rm{n}}{\rm{t}}{\rm{a}}{\rm{g}}{\rm{e}}=\frac{{\rm{N}}{\rm{o}}.\,{\rm{o}}{\rm{f}}\,{\rm{d}}{\rm{e}}{\rm{a}}{\rm{t}}{\rm{h}}{\rm{s}}\,{\rm{a}}{\rm{f}}{\rm{t}}{\rm{e}}{\rm{r}}\,15\,{\rm{d}}{\rm{a}}{\rm{y}}{\rm{s}}\,}{{\rm{T}}{\rm{o}}{\rm{t}}{\rm{a}}{\rm{l}}\,{\rm{p}}{\rm{o}}{\rm{p}}{\rm{u}}{\rm{l}}{\rm{a}}{\rm{t}}{\rm{i}}{\rm{o}}{\rm{n}}}\times 100$$

## Conclusions

The probable PE-DPs generated due to biodegradation of polythene by *Aspergillus terreus* strain MANF1/WL and *Aspergillus sydowii* strain PNPF15/TS were detected using GC-MS analysis (2,Naphthalene carboxylic acid; Diethylpthalate; 1,2-Bis(trimethylsilyl) benzene; Hexasiloxane; 2-Cyclohexen-1; Dibutyl phthalate; 1,2 Benzenedicarboxylic acid; 7-Methylenebicyclo [3.2.0] hept-3-en-2-one; Octadecanoic acid; Cyclohexane-1 and Dodecahydropyrido [1,2-b] isoquinolin-6-one). Further, PE-DPs of the both the fungi didn’t reported any significant toxic effect on both plant and animal systems. To commercialize the polythene degradation using fungi efficiently, the polythene degrading enzymes needs to be fished out and characterized.

## Supplementary information

Supplementary information for Gas chromatography-Mass Spectra analysis and deleterious potential of fungal based polythene-degradation products

## Data Availability

Data would be available on request to corresponding author.
