# Supplementary material for: Gas chromatography-Mass Spectra analysis and deleterious potential of fungal based polythene-degradation products
Source: Sci Rep. 2019 Feb 7;9:1599. doi: 10.1038/s41598-018-37738-6 (PMC6367317; doi:10.1038/s41598-018-37738-6)
Supplement: Supplementary file 1 — Supplementary information for Gas chromatography-Mass Spectra analysis and deleterious potential of fungal based polythene-degradation products [file 41598_2018_37738_MOESM1_ESM.docx]

Supplementary information for

**Gas chromatography-Mass Spectra analysis and deleterious potential of fungal based polythene-degradation products**

Manisha K. Sangale^1^, Mohd. Shahnawaz* ^1,2^, and Avinash B. Ade*^1^

^1^Department of Botany, Savitribai Phule Pune University, Pune-411007, Maharashtra, India.

^2^Present address, Plant Biotechnology Division, CSIR-Indian Institute of Integrative Medicine, Canal Road Jammu, Jammu-180001, Jammu and Kashmir, India

*Corresponding authors: Phone: +91-020-25601439. Fax: +91-020-25690498.

Email: mskhakii@unipune.ac.in

avinashade@unipune.ac.in

| Supplementary Table S1. List of the identified compounds produced due to degradation of polythene using *Aspergillus terreus* strain MANF1/WL at pH 9.5 and *Aspergillus sydowii* strain PNPF15/TS at pH 3.5 | | | | | | |
| --- | --- | --- | --- | --- | --- | --- |
| Sr.  No. | RT | Name of the compound | pH 3.5 | | pH 9.5 | |
|  |  |  | Control | PEDP *Aspergillus sydowii* strain PNPF15/TS | Control | PEDP of *Aspergillus terreus* strain MANF1/WL |
| 1 | 2.32 | Propane |  |  |  |  |
| 2 | 2.47 | Pentanoic acid |  |  |  |  |
| 3 | 4.40 | 2 Naphthalene carboxylic acid |  |  |  |  |
| 4 | 4.90 | 7-Methylenebicyclo[3.2.0] hept-3-en-2-one |  |  |  |  |
| 5 | 4.91 | 1,3,5 Cycloheptatriene |  |  |  |  |
| 6 | 6.056 | Trimethylsilyl methanol |  |  |  |  |
| 7 | 8.84 | Beta-ocimene |  |  |  |  |
| 8 | 12.25 | 2-Butoxyethylacetate |  |  |  |  |
| 9 | 13.95 | Levomethenol |  |  |  |  |
| 10 | 15.80 | Dodecane |  |  |  |  |
| 11 | 18.19 | 2-Cyclohexen -1 |  |  |  |  |
| 12 | 21.02 | Diethylpthalate |  |  |  |  |
| 13 | 21.84 | Cyclooctasiloxane |  |  |  |  |
| 14 | 22.54 | Octadecane |  |  |  |  |
| 15 | 25.934 | Dibutyl phthalate |  |  |  |  |
| 16 | 27.14 | Cyclohexane-1 |  |  |  |  |
| 17 | 29.12 | 1,2-Bis(trimethylsilyl)benzene |  |  |  |  |
| 18 | 29.73 | Hexasiloxane |  |  |  |  |
| 19 | 28.01 | 1,4-Benzenediol, |  |  |  |  |
| 20 | 28.45 | Dodecahydropyrido[1,2-b] isoquinolin-6-one |  |  |  |  |
| 21 | 32.615 | 1,2 Benzenedicarboxylic acid |  |  |  |  |
| 22 | 32.642 | Hexadecanoic acid |  |  |  |  |
| 23 | 36.09 | Cyclodecasiloxane |  |  |  |  |
| 24 | 38.86 | Octasiloxane |  |  |  |  |
| RT: Retention time | | | | | | |

| Supplementary Table S2. Effect of PEDP of fungal isolates on percent seed germination of Sorghum seed | | | | | |
| --- | --- | --- | --- | --- | --- |
| Treatment | Conc. | Percent seed germination | | | Average |
|  |  | R1 | R2 | R3 |  |
| Distilled Water | Control | 100.00 | 85.00 | 100.00 | 95.00±8.66^a^ |
| PEDP of *Aspergillus terreus* strain MANF1/WL | 10% | 085.00 | 80.00 | 090.00 | 85.00±50^a^ |
|  | 25% | 100.00 | 90.00 | 085.00 | 91.67±7.64^a^ |
|  | 50% | 080.00 | 95.00 | 090.00 | 88.33±7.64^a^ |
| PEDP of *Aspergillus sydowii* strain PNPF15/TS | 10% | 090.00 | 85.00 | 090.00 | 88.33±2.89^a^ |
|  | 25% | 080.00 | 95.00 | 090.00 | 87.37 ±7.64^a^ |
|  | 50% | 095.00 | 80.00 | 085.00 | 84.67±7.64^a^ |
| a: no significant difference at 0.05 level of significance | | | | | |

| Supplementary Table S3. Effect of PEDP of fungal isolates on elongation inhibition rate of Sorghum seed | | | | | |
| --- | --- | --- | --- | --- | --- |
| Treatment | Conc. | Elongation inhibition rate | | | Average |
|  |  | R1 | R2 | R3 |  |
| Distilled water | Control | 0.00 | 0.00 | 0.00 | 00.00±00^c^ |
| PEDP of *Aspergillus terreus* strain MANF1/WL | 10% | 8.16 | 18.15 | 8.67 | 11.66±5.62^b^ |
|  | 25% | 35.98 | 19.43 | 43.89 | 33.10±12.48^a^ |
|  | 50% | 42.87 | 29.70 | 31.66 | 34.75±7.10^a^ |
| PEDP of *Aspergillus sydowii* strain PNPF15/TS | 10% | 1.824 | 2.7 | 2.65 | 2.39±0.49^bc^ |
|  | 25% | 0.98 | 1.31 | 1.64 | 1.47 ±0.16^c^ |
|  | 50% | 2.49 | 3.105 | 3.72 | 3.10±0.615^bc^ |
| Different small case letter over the values are significantly different at 0.05 level of significance | | | | | |

| Supplementary Table S4. Effect of PEDP of Fungal isolates on germination index of Sorghum seeds | | | | | |
| --- | --- | --- | --- | --- | --- |
| Treatment | Concentration | germination index | | | Average |
|  |  | R1 | R2 | R3 |  |
| Distilled water | Control | 100.00 | 100.00 | 100.00 | 100±0.00^ab^ |
| PEDP of *Aspergillus terreus* strain MANF1/WL | 10% | 82.10 | 62.56 | 57.94 | 67.53±12.82^b^ |
|  | 25% | 65.96 | 119.10 | 64.85 | 83.30±31.01^ab^ |
|  | 50% | 74.44 | 63.03 | 67.09 | 68.19±5.79^b^ |
| PEDP of *Aspergillus sydowii* strain PNPF15/TS | 10% | 73.63 | 83.60 | 127.85 | 95.03±28.86 ^ab^ |
|  | 25% | 89.82 | 139.28 | 107.70 | 112.27±25.04^a^ |
|  | 50% | 119.89 | 57.49 | 92.07 | 89.82±31.26^ab^ |
| Different small case letter over the values are significantly different at 0.05 level of significance | | | | | |

| Supplementary Table S5. Mortality rate of fishes after 15 days of incubation in different concentrations of PEDP produced by the fungal isolates | | | | | |
| --- | --- | --- | --- | --- | --- |
| Treatment code | Tank No. | PEDP volume(ml) | Aquarium volume (L) | Concentration of PEDP (%) | Mortality rate (%) |
| Control | C | 00.00 | 8 | 0.00 | 0.00 |
| PEDP of *Aspergillus terreus* strain MANGF1/WL | 1 | 08.00 |  | 0.10 | 0.00 |
|  | 2 | 40.00 |  | 0.50 | 0.00 |
|  | 3 | 80.00 |  | 1.00 | 0.00 |
| PEDP of *Aspergillus sydowii* strain PNPF15/TS | 4 | 08.00 |  | 0.10 | 0.00 |
|  | 5 | 40.00 |  | 0.50 | 0.00 |
|  | 6 | 80.00 |  | 1.00 | 0.00 |
